# Factorial Analysis of Fiber Laser Fusion Cutting of AISI 304 Stainless Steel: Evaluation of Effects on Process Performance, Kerf Geometry and Cut Edge Roughness

**DOI:** 10.3390/ma14102669

**Published:** 2021-05-19

**Authors:** Achim Mahrle, Madlen Borkmann, Peer Pfohl

**Affiliations:** 1 Fraunhofer IWS Dresden, Winterbergstraße 28, D-01277 Dresden, Germany; madlen.borkmann@iws.fraunhofer.de; 2 Institute of Manufacturing Science and Engineering, TU Dresden, D-01062 Dresden, Germany; peer.pfohl@tu-dresden.de

**Keywords:** fiber laser fusion cutting, AISI 304 stainless steel, design-of-experiments, gas flow simulation, kerf geometry, roughness evaluation

## Abstract

Factorial Design-of-Experiment analyses were applied for conventional and beam oscillation fiber laser cutting of 10 mm thick AISI 304 stainless steel. Considered factors in case of the conventional process with a static beam involve both laser and cutting gas parameters, in particular the laser power, the focal plane position, the cutting gas pressure, the nozzle stand-off distance as well as the nozzle diameter. The conducted trials were evaluated with respect to the achievable cutting speed, the cut kerf geometry and the cut edge roughness. Noticeable correlations between cut edge roughness and cut kerf geometry stimulated the development of a corresponding Computational Fluid Dynamics (CFD) model of the cutting gas flow through the kerf. A specific approach of data synchronization revealed that the experimentally determined roughness values do well correlate with numerically computed values of the backward directed component of the gas-induced shear stress and that the cut kerf geometry as internal process-inherent boundary condition influences relevant cutting characteristics more than controllable external cutting gas parameters. Finally, effects of circular beam oscillation were investigated by an additional factorial analysis considering the laser power, the focal plane position, the oscillation frequency and the oscillation amplitude as factors. The results demonstrate the potential of beam oscillation techniques for quality improvements in laser cutting.

## 1. Introduction

**Figure 1:**
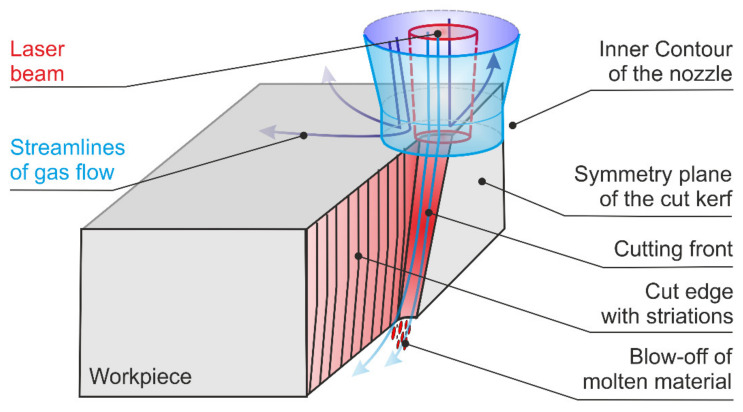
Schematic illustration of the laser fusion cutting process.

Laser fusion cutting relies on the combined action of a focused laser beam and a commonly coaxially arranged high-pressure gas jet. While the laser beam melts the material, the pressure gradients and the shear stress of the gas jet blow the molten metal out of the cutting zone. As a result, a cut kerf of particular shape and size is generated that separates both edges of the remaining material along the desired cut contour. Figure 1 shows a schematic drawing of the laser beam fusion cutting process. Because of the use of nitrogen as a cutting gas, laser fusion cutting does not involve any chemical reaction as in oxygen cutting, and the melting process relies on the beam-matter interaction only [1,2]. 

Despite the fact that the operating principle of laser cutting is quite simple, there is still a lack of a profound understanding of the involved physical mechanisms and inherent interactions that eventually determine the technical performance indicators of the cutting process, i.e., the achievable cutting speed, the cut kerf shape and size, the cut edge roughness and the amount of dross attachment. Recent research work in this field was primarily triggered by the advent of high-power and high-brightness solid-state lasers, i.e., fiber and disk lasers. Indeed, the detected differences between the emerging solid-state laser cutting with fiber and disk lasers with emission wavelengths of about 1 µm and the well-established and industrially proven laser cutting with CO_2_ lasers with an emission wavelength of 10.6 µm brought the study of laser fusion cutting back into the focus of scientific research interest.

One of the first experimental investigations on this topic was performed by Wandera et al. [3] who compared disk, fiber and CO_2_ laser cutting results on stainless steel in a thickness range between 1 and 6 mm. They reported that the solid-state laser sources are capable of cutting thin-section sheets much faster than the CO_2_ laser but give rise to a different cut edge topography with higher values of surface roughness in thicker-section sheets. These principal characteristics were also confirmed by several subsequent studies. Himmer et al. [4] compared quality and performance of CO_2_ and fiber laser cutting in a thickness range between 1 and 10 mm. The achieved higher cutting speeds in fiber laser cutting were reasoned by higher absorption rates and better focus ability as a result of the shorter wavelength of fiber laser radiation. Hilton [5] described a series of experiments of cutting stainless steel plates from 0.6 to 6 mm in thickness using a disk and a CO_2_ laser both operating at 5 kW power. It was shown that the disk laser was capable of cutting thin materials at higher speed and with lower edge roughness than the CO_2_ laser but produced lower cut quality in terms of surface roughness for 3 and 6 mm thickness material. Scintilla et al. [6,7,8] performed a comparative study on fusion cutting cold work steels with 1, 5 and 8 mm in thickness using disk and CO_2_ laser beams of similar geometry in terms of focal diameter and depth of focus (Rayleigh length). The finding of much higher cutting rates for the disk laser in 1 mm thickness sheets was considered to be indicative for the primary effect of an increased absorption at the shorter wavelength. However, this advantage was found to diminish with increased sheet thickness. This peculiar dependence could be theoretically reasoned by the pronounced dependence of the absorptivity on the angle of incidence according to the Fresnel equations [9,10,11,12,13,14]. It became obvious that a relatively low value of the Brewster angle of about 80° for maximized energy absorption in case of 1 µm wavelength might be a drawback for solid-state lasers because corresponding values of the cut front inclination are typically higher than this value for thick-section cutting. In case of 10.6 µm of the CO_2_ laser, a theoretical Brewster angle of about 87° seems to be matching perfectly under those conditions. Besides those energetic constraints, it was also argued that the cutting regime in thick-section sheets could be limited not by the energy consumption but by the melt ejection through the narrow cut kerf [15]. Sparkes et al. [16,17] identified two distinct melt-eject failure mechanisms in fiber laser cutting of medium-section stainless steel in the range of 6–10 mm thickness. First, the boundary layer separation of the cutting gas flow from the cutting front resulted in additional melting and dross attachment at the base of the cut. Second, melt generated at the top of the cut could not be forced down through the kerf and caused internal melt circulation and additional melting through increased residence times. Wandera and Kujanpää [18] theoretically estimated melt removal rates for thick-section stainless steel laser cutting and showed that particularly the assist gas pressure, the nozzle diameter and the focal point position affect the efficiency of melt removal from the cut kerf. Despite the reported difficulties in obtaining full melt ejection through narrow kerfs in thick-section cutting, dross-free cut edges with acceptable surface roughness were achieved by an adequate control of beam, gas and process parameters, e.g., Wandera and Kujanpää [19] optimized parameters for fiber laser cutting of stainless steel plates with 10 mm in thickness and achieved surface roughness values R_Z,Max_ ≈ 80 µm for particular parameter combinations. Goppold et al. [20] applied different optical setups and beam geometries and adjusted gas pressures, focal plane positions and gas nozzle diameters to achieve acceptable cut edge qualities in fiber laser cutting stainless steel in a thickness range between 1 and 15 mm. In case of the 10 mm thick probes, a surface roughness of R_Z_,_Max_ ≈ 70 µm was recorded. Later, cut quality enhancements were reported for fiber laser cutting mild and stainless steels in 12 mm thickness by use of beam oscillation methods [21]. Pang and Haecker [22] introduced optical setups with annular intensity distributions for disk laser cutting and achieved burr-free cuts in 10 mm stainless steel. However, corresponding roughness values were not mentioned.

Despite these achievements, burr-free solid-state laser cut edges still clearly differ in visual appearance from those in CO_2_ laser fusion cutting and give typically rise to higher values in terms of surface roughness, particularly for inert-gas stainless steel cutting with sheet thicknesses above 6 mm. A key feature of the cut edge topography is melt striations, and the investigation of their origin, shape and size is still within the focus of scientific research and involves both experimental and theoretical studies. Hirano and Fabbro [23] observed the striation generation process in inert-gas laser cutting of 3 mm thick mild steel sheets and found instabilities of the melt flow in regions of the kerf front and the cut kerf flank. They concluded that the instability in the side region causes the periodic initiation of cut edge striations. Inherent dependencies on laser wavelength as well as processing parameters were also discussed in detail [24,25]. Ermolaev et al. [26] used the trim-cut method to visualize the melt flow in inert-gas cutting stainless steel sheets of 6 mm thickness by CO_2_ and fiber laser radiation and found that the type of radiation, i.e., the laser wavelength, influences the liquid melt flow behavior on the cut front. They reported a melt flow destabilization in case of fiber laser cutting. Arntz et al. [27] also analyzed the melt flow dynamics by using the trim-cut technique and in situ high-speed video-diagnostics and found that the occurrence of unstable melt streams directly correlates with increased surface roughness. They also reported an effect of multiple reflections on the striation pattern in case of laser fusion cutting with 1 µm wavelength [28]. Petring [29] reported a correlation of the cut flank roughness with theoretically calculated cutting front curvatures as well as a negative correlation of the dross height with the calculated maximum penetration of the supersonic gas jet into the kerf. Amara et al. [30] performed a Computational Fluid Dynamics (CFD) study of the molten film dynamics and investigated the effect of wavelength on temperature distribution, cut front shape and kerf formation. They found higher maximum temperatures, steeper cutting fronts and smaller striations in case of the CO_2_ laser wavelength. Recently, the same research group developed a comprehensive numerical model of the laser beam cutting process which accounts for laser absorption, phase transition, heat and mass transfer, fluid flow, kerf formation and gas jet flow [31].

It is important to keep in mind that the gas flow does not only causes melt ejection failures in case of insufficient flow rates but also affects the striation formation and cut edge roughness. The purpose of the gas flow is to blow the molten material out from the kerf, and thus, it is quite obvious to assume a strong impact of gas characteristics on striation formation and resultant cut edge topography. However, due to the impossibility to visualize those interactions directly and the difficulty to consider all of the relevant effects in corresponding models, gas flow aspects were usually investigated separately. A first comprehensive characterization of the cutting gas flow was given by Fieret [32,33]. Amongst others, a direct correlation between gas pressure and achievable cutting speeds was found. In a further fundamental work by Petring et al. [34] gas dynamic effects within kerfs were approximated by means of modelled kerf channels made of transparent materials that enabled a Schlieren analysis of the gas flow. Since then, much research on cutting gas characteristics, including investigations of different nozzle designs and nozzle arrangements, has been conducted, e.g., Brandt et al. [35,36] investigated the effects of nozzle orientation on the gas dynamics of inert-gas laser cutting of mild steel with 1–4 mm in thickness and reported a 50% increase in maximum cutting speed at particular inclination angles of the used tilted off-axis nozzle. Chen et al. [37] studied gas dynamics effects on cut quality in terms of roughness, dross attachment, and recast layer thickness for laser cutting mild steel sheets of 1.6 mm thickness. It was shown that the cut quality varies with gas pressure and nozzle stand-off distance. Man et al. [38] reported results from investigations on effects of inlet gas pressure, nozzle stand-off distance, cut kerf width and depth upon the gas jet patterns inside cut kerf models by using the shadow graphic technique. A review on published other works relating to the crucial role of the assist gas in laser beam cutting was recently compiled by Riveiro et al. [39].

Besides these useful insights into the complex inherent mechanisms of laser beam fusion cutting, it becomes particularly important from a practical point of view to elaborate how the controllable beam and gas jet parameters have to be adjusted to get optimized processing results for a given material and thickness. The problem in this kind of endeavor consists in the multitude of influencing variables and the highly expectable presence of interactions between them. Hence, the use of Design-of-Experiments (DoE) methods seems to be indispensable to meet this challenge. These methods offer tailored designs for factor screenings, factor weighting and revealing of factor–factor interactions by use of fractional and full two-level factorials, as well as for regression modeling of nonlinear functional dependencies and optimization by use of response surface methods [40,41]. Indeed, DoE methods were already successfully tested in studies on laser beam cutting, e.g., Son and Lee [42] investigated CO_2_ laser cutting of structural and stainless steel with 2 mm in thickness and applied multiple regression analysis to describe correlations between processing parameters and cutting quality. Huehnlein et al. [43] reported on the optimization of laser cutting thin alumina layers based on factor screening and response surface designs. Eltawahni et al. [44] applied DoE methods to relate cutting edge quality quantities to process parameters in CO_2_ laser cutting of medical grade AISI 316L stainless steel with a thickness of 2 mm. Tahir and Aquida [45] demonstrated the identification of optimum parameter ranges for CO_2_ laser cutting of 22MnB5 boron steel of 1.7 mm thickness by use of a response surface design. Kechagias et al. [46] applied a full factorial experimental methodology to analyze surface quality characteristics of 3D printed Polyactic Acid (PLA) plates with 4 mm in thickness cut by use of a CO_2_ laser. Sharma and Yadava [47] combined Taguchi-based experimental designs and grey relational analysis (GRA) to optimize thin sheet Neodymium-doped Yttrium Aluminium Garnet (Nd:YAG) laser cutting of Ni-based super-alloy with consideration of multiple performance characteristics. A general review on optimization techniques in metal cutting processes was given by Mukherjee and Ray [48].

In the present study, two-level factorial designs are applied to reveal the most vital factors in laser beam fusion cutting of AISI 304 stainless steel with 10 mm thickness. By combining work on experimental laser beam cutting and numerical simulation of cutting gas characteristics, also a light is shed upon the role of inherent processing variables. Furthermore, the significance of oscillation parameters in cutting with circular dynamic beam shaping is evaluated in comparison to static beam parameters.

## 2. Materials and Methods

Cutting experiments were conducted on sheets of AISI 304 stainless steel (Walzwerke Einsal GmbH, Nachrodt, Germany) with a thickness of 10 mm. The detailed chemical composition of the test material is given in Table 1.

**Figure 2:**
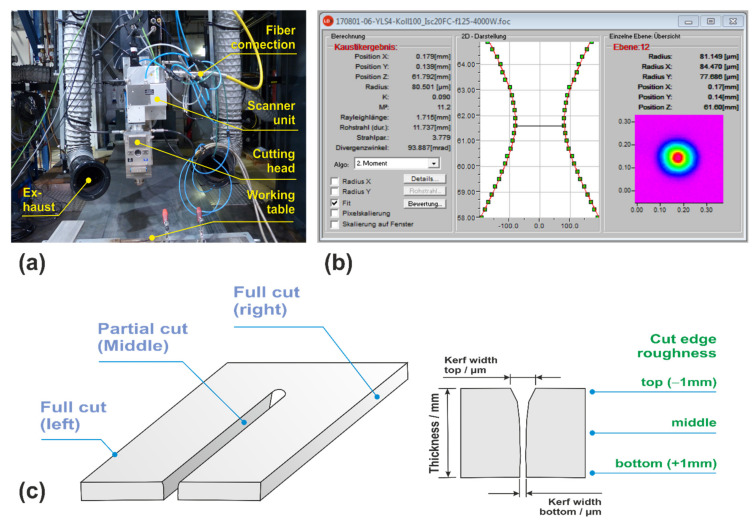
Experimental setup for the conducted cutting trials (**a**), measured beam caustics (**b**) and schematic of the produced cutting probes (**c**).

A 4 kW multimode fiber laser IPG YLR 4000 (IPG Laser GmbH, Burbach, Germany) was used as the beam source in combination with a Precitec HP-SSL cutting head (Precitec GmbH & Co. KG, Gaggenau, Germany). The beam coming out of the delivery fiber with a diameter of 100 µm was collimated by a 100 mm lens and then focused by a lens with 125 mm focal length. The resultant beam caustic was measured with the Primes MicroSpotMonitor tool (Primes GmbH, Pfungstadt, Germany). The measurement indicates an actual focal beam radius of about 80 µm and a Rayleigh length or depth of focus of about 1.7 mm, respectively. The use of such a beam with short Rayleigh length and small spot size can be considered as a typical feature of laser beam cutting with beam oscillation, in which high local beam intensities are desirable to achieve an improved process performance in comparison to conventional laser cutting with a static beam and typically larger spot sizes and Rayleigh lengths. The selected optical configuration can be considered as a standard setup for laser beam oscillation cutting. The scanning unit ScanLab IntelliScan 20 FC (SCANLAB GmbH, Puchheim/München, Germany) was used for the purpose of lateral two-dimensional beam oscillation. In combination with the specified optical setup, this device enables oscillation amplitudes of the focal spot in the range of up to 100 µm in a frequency range of up to 4 kHz. The experimental setup as applied for the cutting trials without and with beam oscillation is shown in Figure 2. Nitrogen was used as a cutting gas in combination with a nozzle of the conical standard type. Considered factors as controllable independent variables of the cutting process included the laser power, the focal plane position (position of the beam waist in relation to the cutting probe), the cutting gas pressure, the nozzle stand-off distance (distance between nozzle outlet and top surface of the cutting probe), the nozzle diameter, the beam oscillation pattern, the oscillation frequency and the oscillation amplitude. Cutting test samples were produced for each of the considered parameter sets by three successive cuts on base material stripes of 100 mm width. The first and the third cut were carried out as cuts over the full width of the stripes and the second one as partial cut over a length of 50 mm. The resultant test samples with a length of 100 mm (i.e., the width of the original base material stripes), a width of 20 mm and a thickness of 10 mm allow for an assessment of the left and right cut edges as well as a measurement of geometrical cut kerf features.

Roughness values as common evaluation criterion of cut edge quality were determined as result of profile (line) measurements with a Jenoptik Hommel-Etamic T100 wave instrument (JENOPTIK AG, Jena, Germany) on both cut edge sides as R_a_ (arithmetical mean deviation of the assessed profile) and R_z_ (average distance between the highest peak and lowest valley) values at different vertical positions of the cut edge as indicated on the right-hand-side in Figure 2. The cut kerf geometries are characterized in terms of kerf width at the top and bottom surface of the test probes measured with a Keyence VHX-5000 digital microscope (KEYENCE DEUTSCHLAND GmbH, Neu-Isenburg, Germany). This device was also used for an evaluation of the topographic cut edge structure as a whole.

The cutting performance is evaluated in terms of the cutting speed limit, i.e., the achievable maximum cutting speed for a complete cut through the 10 mm thick base material. Preparatory cutting trials with a linear increase of cutting speed with beam spot position up to the point of the process breakdown were conducted to determine this value. The detected cutting speed was confirmed in a second step by performing a full length cut, and eventually used to produce the defined test sample for the particular parameter constellation. 

The treatment of the cutting speed as a response of particular parameter constellations is of essential importance for the performed study and the assessment of the results. In general, the cutting speed can also be and even is often used as independent process variable to control the cut edge quality. In particular, the attachment of dross at the lower cut edge is found to be affected by the cutting speed. As a rule of thumb, a cutting velocity of about 80% of the cutting speed limit gives rise to the best cut edge quality whereas values above and below this optimum make both the quality worse. Most important is the point that the optimum cutting speed is always related to the cutting speed limit. Consequently, within a parameter field including different combinations of independent factors that are all initially anticipated to have an impact on the cutting speed limit, it is not reasonable to define fixed levels of cutting speed. Therefore, the cutting speed was considered as a response of the experiments. This makes the cut edge qualities in terms of roughness comparable because they can be regarded in any case as the cut edge qualities at the cutting speed limit. With respect to the purpose of this study, this is not a drawback because the primary aim of this study was the identification of most vital factors and possible interactions but not the optimization of the process. 

**Figure 3:**
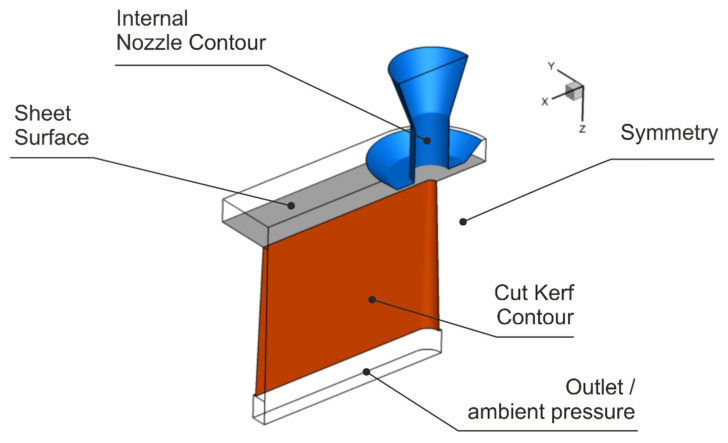
Geometrical model of the cutting gas flow.

Additional simulations of the cutting gas flow through the cut kerf were conducted to support the interpretation of the experimental data. For that purpose, a corresponding Computational Fluid Dynamics (CFD) model was developed by use of the commercial software package Ansys Fluent (Version R2, 2019, ANSYS, Inc., Canonsburg, PA, USA). The parametrized model allows for variations of gas pressure, nozzle stand-off distance and diameter, and cut kerf geometry to investigate the impact of these factors on resultant shear-stress distributions on cutting front and cut kerf edges. For the sake of simplicity, the cut edges of the geometrical model are approximated by inclined but even planes, and partial shell surfaces of truncated cones are used to model the cut front, both in accordance with the experimentally measured kerf width values at the top and the bottom surface of the sheet. The physical model solves the conservation equations of energy, mass and momentum of a turbulent flow under steady-state conditions taking the viscous Shear Stress Transport (SST) k-ω turbulence approach into account. Nitrogen as used cutting gas is considered as an ideal gas with the cutting gas pressure p_G_ specified at the nozzle inlet according to the corresponding experiments and an ambient pressure p_∞_ of 0.1 MPa. No-slip adiabatic boundary conditions are applied for all solid boundaries. The computational domain of the model is schematically shown in Figure 3.

The used designs for both the experimental and numerical investigations were prepared and analyzed by use of the Design-of-Experiment (DoE) software tool DesignExpert (Version 11, 2017, STAT-EASE, Inc., Minneapolis, MN, USA) that also allowed for the randomization of the running sequence. In case of the conventional cutting process and the CFD simulation, a regular fractional two-level factorial design of resolution 2^5−1^ for the considered 5 factors with corresponding 16 different tested parameter constellations was applied, properly supplemented by 5 additional runs at the center point of the design space for curvature check and pure error estimation. In case of the cutting process with beam oscillation and only 4 considered factors, the full factorial design, with 2^4^ = 16 possible parameter constellations and again 5 additional runs at the center point, was applied. With respect to the main purpose of the performed analysis, i.e., the unveiling of the most vital factors and first-order interactions, it is not necessary to detail the corresponding regression models as well as the statistical outcomes and characteristics of the analysis of variance (ANOVA). Therefore, the discussion of the results will remain focused on the qualitative analysis of the results from an engineering point of view. For that purpose, factors and relevant factor–factor interactions with significant effect on the considered responses were selected graphically by means of half-normal probability plots. Based on these selections, regression models were built on a statistical methodology to describe the dependencies between effects and factors. In some cases, data transformations including inverse, logarithmic or exponential approaches were considered to improve the quality of the models. The effects of significant models’ terms are discussed by means of the resultant model graphs. 

## 3. Results and Discussion

The whole structure of the performed work of this study consists of three primary parts. First, experimental results of the factorial analysis of conventional laser cutting with a static beam are presented. Considered factors are the laser power P_L_, the focal plane position z_FP_, the gas pressure p_G_, the nozzle diameter d_N_ and the nozzle stand-off distance Δz_N_. Second, the numerical results of the CFD study of the cutting gas flow characteristics are shown. For this part, a specific approach of data synchronization was for the first time applied with the purpose to link the results of the performed computational analysis to the outcomes of the experimental part. The derived approach simply bases on the intuitive idea to use the same design of factor–factor combinations as in the experimental study but to address the effects of the beam parameters laser power and focal plane position on the gas flow in a virtual way by adjusting the geometrical model of the CFD analysis according to the corresponding measured values of kerf widths at the top and bottom surface of the sheets being cut. This allowed for using identical parameter constellations for both the experimental trials and the gas flow simulations and enabled a correlation analysis between different outcomes of both studies. It will be shown later that this approach is sufficiently justified by the results of this work. Finally, the laser cutting process with circular beam oscillation is analyzed by an adapted factorial design. Here, the laser power P_L_, the focal plane position z_FP_, the oscillation amplitude a_0_ and the oscillation frequency f_0_ were involved as independent variables or factors, respectively. Using the same levels of laser power and focal plane position as before in the experimental design of conventional laser cutting, the influence of oscillation parameters can be evaluated against the principal parameters of the static beam. 

### 3.1. Factorial Analysis of Conventional Cutting 

The experimental design of the factorial analysis of conventional laser cutting with a static beam and already randomized run order is listed in Table 2.

The used levels (low and high) of the considered factors laser power P_L_ (Factor A), focal plane position f_FP_ (Factor B), cutting gas pressure (Factor C), nozzle stand-off distance Δz_N_ (Factor D) and nozzle diameter d_N_ (Factor E) were determined in pre-investigations to ensure that full cuts through the 10 mm thick sheets are achievable for each of the defined factor combinations. In addition to the 16 regular runs of the fractional factorial design with resolution 2^5−1^ also 5 replications of the design space center point configuration were added with the corresponding factor values P_L_ = 3.5 kW, f_FP_ = 5 mm, p_G_ = 1.6 MPa, Δz_N_ = 0.75 mm and d_N_ = 3.0 mm. Here, the focal plane position is always defined with respect to the position of the underside of the sheet that indicates the origin or zero position of the used coordinate system. Variations of the nozzle stand-off distance as magnitude of the distance between the nozzle outlet and the top surface of the sheet were considered for the adjustment of the focal plane position as well. The focal plane position determines the beam size at the top surface of the sheets being cut. By means of the beam propagation equation

(1) $$d_{\mathrm{Beam}}\left(z\right)=2\times r_{0}\times \sqrt{1+{\left(z/z_{R}\right)}^{2}}$$

and using the measured characteristics focus radius *r*_0_ = 80 µm and Rayleigh length *z*_R_ = 1.7 mm of the applied beam, see Figure 2, the beam diameter *d*_Beam_ can be estimated as a function of focal plane position giving rise to the values of 718, 493 and 282 µm for the considered positions at 2.5, 5.0 and 7.5 mm. 

**Figure 4:**
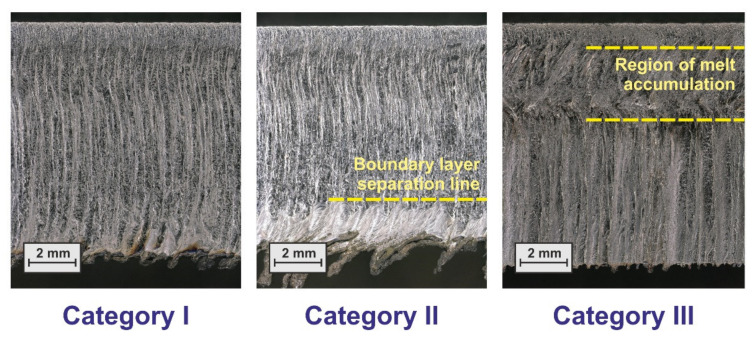
Different cut edge categories for different parameter sets. Category I—Run 07, Category II—Run 01, and Category III—Run 12.

A first visual inspection of the produced test specimen revealed different cut edge categories being shown in Figure 4. Cuts of Category I (Run 07) show an almost regular cut edge structure and only sporadic and moderate dross formation at the lower sheet edge. These cut edges show the characteristic features of standard fiber laser cuts under conditions of almost well-balanced parameter constellations. In contrast, cuts of Categories II and III are characterized by abrupt transitions between different regions of the edge. Here, Category II (Run 01) cuts show the typical features of the boundary layer separation phenomenon with insufficient blow out of the melt in the lower region of the cut edge, whereas cuts of Category III (Run 12) exhibit strong melt accumulations in the upper region of the cut edge. Table 3 indicates the cut edge category for each of the performed cutting trials. It should be noted that cut edges of Category III always resulted for focal plane positions at 2.5 and 5.0 mm, and that for the high focal plane position of 7.5 mm either cuts of Category II or Category I were produced. 

Nevertheless, and despite this appearance of obviously different cutting regimes, the generated data fields of the considered responses show sufficient consistency for a statistically proven effect evaluation. Characteristic values of the raw data distribution, i.e., the minimum and the maximum values, the max/min ratio and the mean values of the measured responses are listed in Table 3. The detected changes are of high technical relevance, and it is worthwhile to clarify where these significant changes primarily result from.

**Figure 5:**
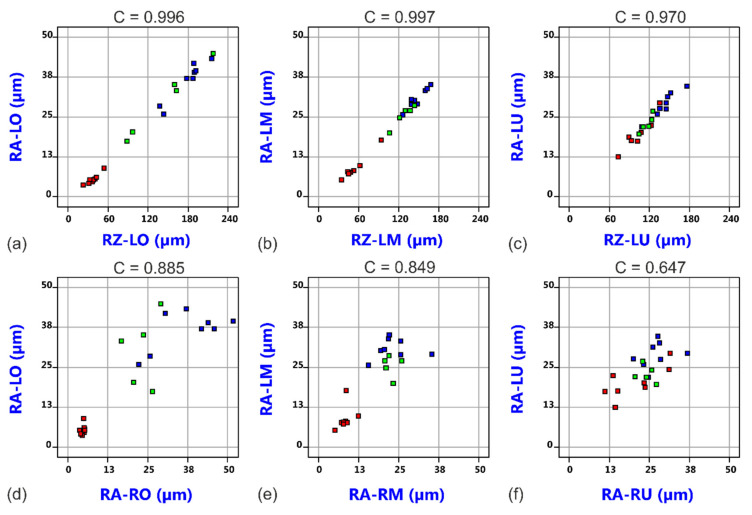
Correlation between R_a_ and R_Z_ roughness values at the top (**a**), the middle (**b**) and the bottom (**c**) measurement positions. Correlation between R_a_ roughness values of the left and right cutting edge at the top (**d**), the middle (**e**) and the bottom (**f**) measurement positions. The data points are colored by value of focal plane position (Blue: 2.5 mm, Green: 5.0 mm, Red: 7.5 mm).

Roughness values were measured at different positions and on both sides of the kerf as R_a_ and R_z_ values. However, as shown in Figure 5, R_a_ and R_z_ data show the anticipatable very high correlation (correlation coefficients between 0.97 and 0.997), and it is also a good correlation between roughness values on both opposite kerf edges (correlation coefficients between 0.647 and 0.885) found. Therefore, only the R_a_ values on the left cut edge—being hereafter denoted as RA-Top (R_a_, top), RA-Middle (R_a_, middle) and RA-Bottom (R_a_, bottom)—are considered in the following analysis but any conclusions concerning effects on cut edge roughness hold true for the other roughness value groups as well.

**Figure 6:**
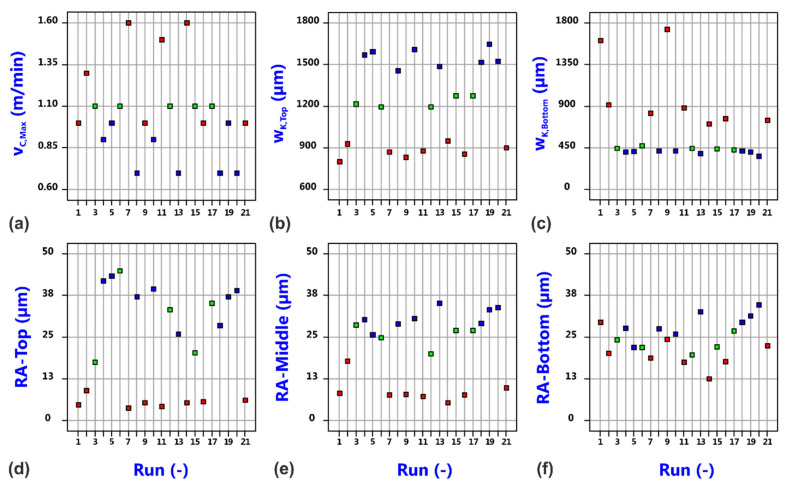
Distribution of raw data of maximum cutting speed (**a**), top kerf width (**b**), bottom kerf with (**c**), top roughness R_a_ (**d**), middle roughness R_a_ (**e**) and bottom roughness (**f**) versus experimental run for laser beam cutting with a static beam. The data points are colored by value of focal plane position (Blue: 2.5 mm, Green: 5.0 mm, Red: 7.5 mm).

Figure 6 shows the raw data distribution versus the run number of the performed cutting trials. The data points for the evaluated responses, i.e., the maximum cutting speed v_C,Max_, the kerf widths w_K,Top_ and w_K,Bottom_ at the top and bottom surface of the sheet, as well as the specified roughness values RA-Top, RA-Middle and RA-Bottom are homogeneously distributed but being colored by the value of the focal plane position, the crucial role of this process variable becomes already obvious. This finding is also confirmed by the detailed analysis of the individual responses.

**Figure 7:**
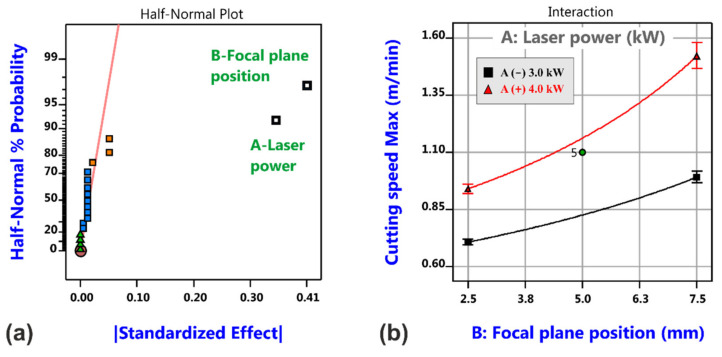
Analysis results of maximum cutting speed for laser beam cutting with a static beam. Half-normal probability plot of cutting speed limit (**a**) and corresponding model graph (**b**).

Figure 7 shows the effect plot and the model graph for the cutting speed limit, i.e., the achievable maximum cutting speed with consideration of an inverse data transformation. Significant factors are the focal plane position and the laser power, both acting in a positive sense, i.e., increasing the focal plane position and/or increasing the laser power will give rise to higher maximum cutting speeds.

**Figure 8:**
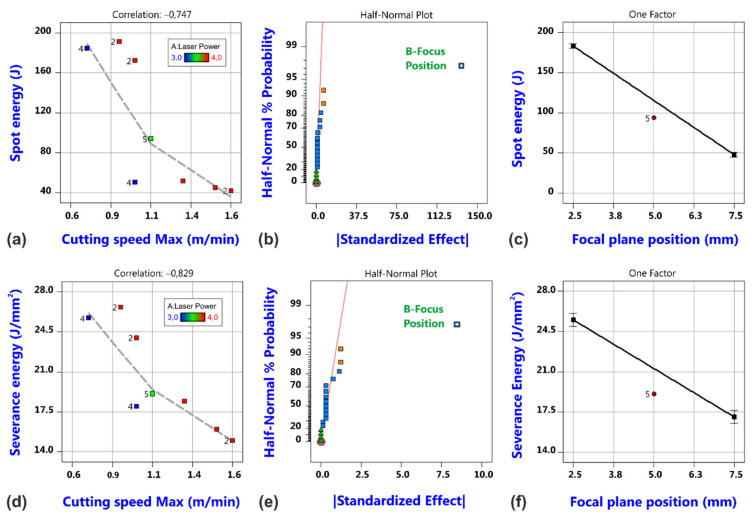
Correlation plot, effect plot and model graph of spot energy (**a**–**c**) and severance energy (**d**–**f**).

As a function of the determined cutting speeds and the applied laser power, some characteristic caloric quantities can be derived such as the spot energy

(2) $$E_{\mathrm{Spot}}=P_{L}\times \Delta t_{\mathrm{WW}}=\frac{P_{L}\times d_{\mathrm{Beam}}}{v_{C,\mathrm{Max}}}$$

by multiplying the applied laser power with the interaction time which is defined here as ratio of beam size *d*_Beam_ at the top surface of the sheet and the determined cutting speed limit [49]. Furthermore, the more common severance energy as used energy per unit cut edge area according to

(3) $$E_{S}=\frac{P_{L}}{v_{C,\mathrm{Max}}\times t_{\mathrm{Sh}}}$$

with the sheet thickness *t*_Sh_ is evaluated [50]. Correlation diagrams with regard to cutting speed as well as effect plots and model graphs for both quantities are shown in Figure 8. In both cases, only the focal plane position is revealed to have a significant effect.

**Figure 9:**
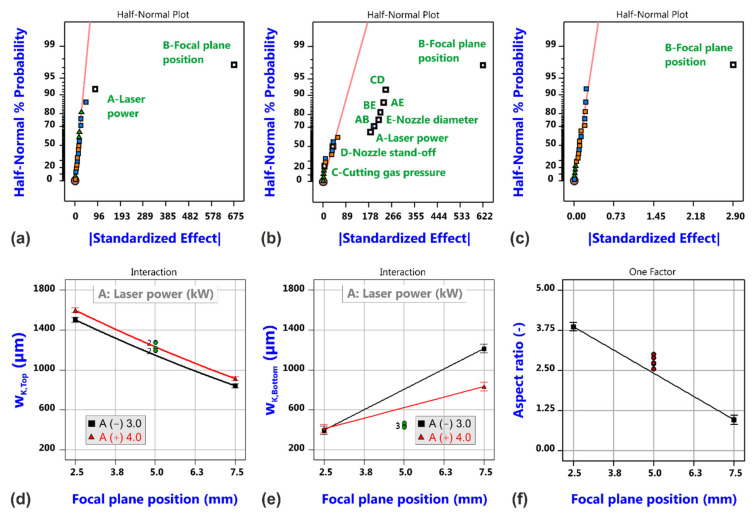
Results of cut kerf geometry analysis for laser cutting with a static beam. Half-normal probability plots of kerf width w_K,Top_ (**a**), kerf width w_K,Bottom_ (**b**) and aspect ratio (**c**), and corresponding model graphs (**d**–**f**). Parameters for model graph (**e**): gas pressure = 1.6 MPa, Nozzle stand-off distance = 0.75 mm, Nozzle diameter = 3.0 mm.

Figure 9 shows the effect plots for the measured geometrical cut kerf quantities, i.e., the kerf width at the top and bottom surface and the corresponding kerf ratio (a–c). It is obvious that the focal plane position is the dominant factor for the kerf geometry, whereas effects of other factors are small (a), moderate (b) or even negligible (c). The laser power shows a marginal effect on the kerf width at the top surface, and as it might expectable, the kerf width slightly increases with laser power (d). In contrast, the model for the kerf width at the bottom of the sheet is quite complex and does not only involve contributions from the individual factors but also shows different interactions (b). The model graph (e) exemplarily shows the interaction between focal plane position z_FP_ and laser power P_L_. The effect of P_L_ is negligible in case of the low level of z_FP_ but noticeable for the high focal plane position. It is further interesting to note that the increase of the bottom kerf width with focal layer position is more pronounced at the low power level. Under this condition, the kerf width at the bottom surface can even become wider than the top kerf width giving rise to aspect ratios < 1. Then again, the cut kerf ratio is simply dominated by the effect of focal plane position only. The physical mechanisms behind these dependencies are expected to be quite sophisticated including a redistribution of laser energy inside the kerf and complex heat and mass flow characteristics of the molten material.

**Figure 10:**
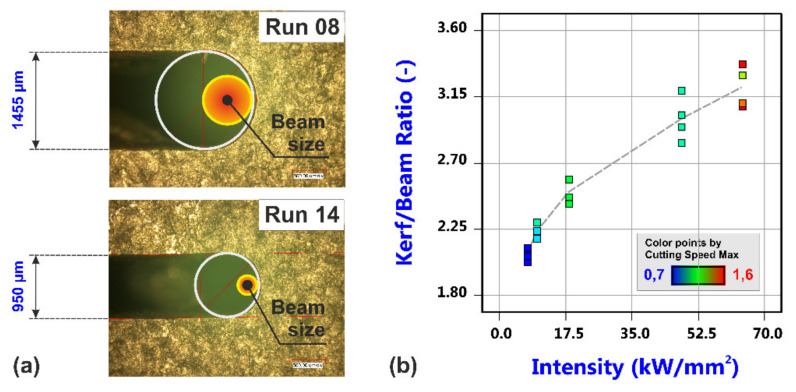
Relation between laser beam size and kerf width at the top surface of the sheet. (**a**) Geometrical conditions for different experimental trials: Run 08 with d_Beam_ = 718 µm and v_C,Max_ = 0.7 m/min, Run 14 with d_Beam_ = 282 µm and v_C,Max_ = 1.6 m/min. (**b**) Kerf/Beam ratio vs. beam intensity with a correlation coefficient of C = 0.964.

Regarding again the kerf width at the top surface of the sheet, the effect of focal plane position was found to be crucial and so it is not far to ask how close the top kerf width matches the diameter of the beam at this particular position. Due to the direct relationship between focal plane position and beam diameter at the top surface, there is of course also a high correlation between the top width and the corresponding beam diameter, but what is particularly interesting to note is the fact that the kerf width is much larger than the beam diameter, as exemplarily shown on the left-hand side of Figure 10. Resultant ratios of the kerf width and the beam size range from 2.03 for a parameter constellation at low focal plane position (Run 8) to 3.37 at high focal plane position (Run 14). As shown on the right-hand side of Figure 10, the ratio shows a high positive correlation to the beam intensity as calculated for the beam size at the top surface despite the fact that also the cutting speed is considerable increased with beam intensity, i.e., from 0.7 m/min for Run 8 (7.41 kW/mm^2^) to 1.6 m/min for Run 14 (64.04 kW/mm^2^). Besides the expectable different thermal states of the directly irradiated zones as a result of the different intensities, the detected high ratios of kerf to beam size do necessarily imply an intensive heat and mass transfer of molten material in lateral direction.

**Figure 11:**
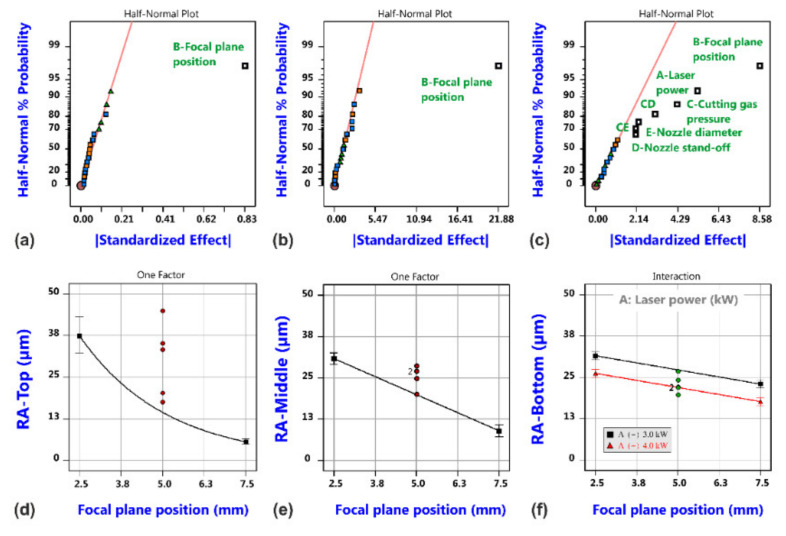
Results of roughness analysis for laser cutting with a static beam. Half-normal probability plots of surface roughness RA-Top (**a**), surface roughness RA-Middle (**b**) and surface roughness RA-Bottom (**c**), and corresponding model graphs (**d**–**f**).

Results of the roughness analysis are shown in Figure 11. The corresponding effect diagrams (a–c) show that the most vital factor is again the focal plane position; this factor is even the only significant one for the roughness values at the top and middle measuring positions. The effect is quite large as shown in the corresponding model graphs (d and e), but this can be regarded to be closely related to the occurrence of different cutting regimes that gave rise to the different cut edge categories according to Figure 4. In both cases, also curvature seems to be significant. This is not the case for the roughness values at the bottom position (f), but more factors and interactions are involved in the corresponding model (c). For a focal position of 7.5 mm and Category I cuts, it is found that the roughness increases from the top to the bottom position, and this is in accordance with the general experience for good quality cuts in fiber laser cutting thick material. 

**Figure 12:**
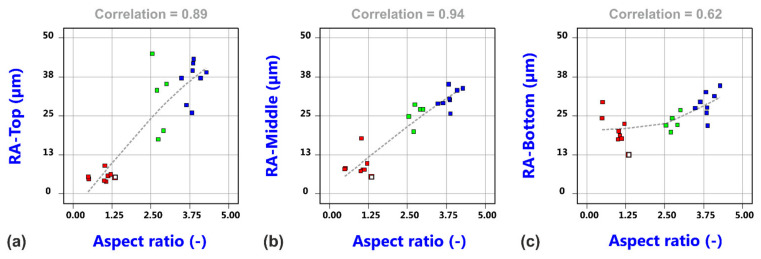
Correlation between roughness values and aspect ratio. (**a**) RA-Top vs. aspect ratio; (**b**) RA-Middle vs. aspect ratio; (**c**) RA-Bottom vs. aspect ratio.

It is surprising that the controllable gas parameters, i.e., the gas pressure, the nozzle diameter and the nozzle stand-off distance, do not show a more pronounced effect on the edge roughness in the investigated parameter ranges. However, noticeable correlations between the roughness values and the aspect ratio were found, as shown in Figure 12. Being aware that the cut kerf geometry also plays a crucial role for the coupling of the cutting gas into the kerf and the resultant cut kerf flow fields, it was obvious to evaluate related effects by corresponding gas flow simulations.

### 3.2. Factorial Analysis of Cutting Gas Flow

**Figure 13:**
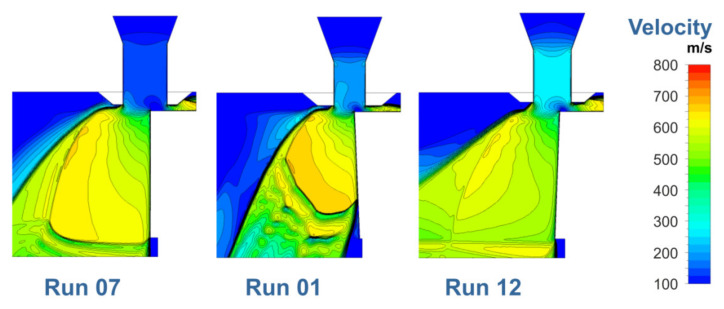
Computed velocity distribution in the symmetry plane for different experimental runs. Parameter constellations are given in Table 2.

**Figure 14:**
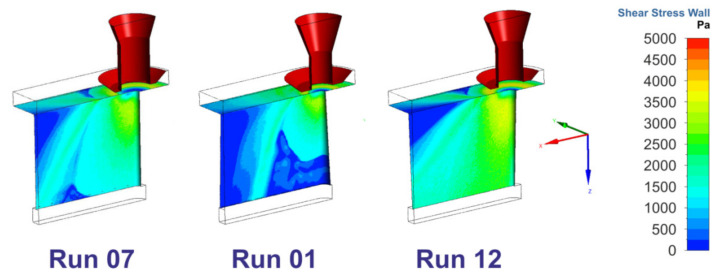
Computed shear stress distribution for different experimental runs. Parameter constellations are given in Table 2.

It is a unique feature of this study that the factorial design of the experimental trials with 21 (16 + 5) tested factor combinations according to Table 2 was also applied to the gas flow simulation. This approach was enabled by an appropriate data synchronization in which the effects of the involved factors on the cut kerf shape and size were considered by adjusting the geometrical model of the CFD simulation according to the measured kerf characteristics for each of the performed individual runs, i.e., the highly sophisticated time- and space-resolved physical phenomena of heating, melting, melt film fluid flow and blow out of the melt were approximated by the final cut kerf geometry. Due to the facts that the melt film is considerably thin in comparison to the whole kerf size and that the blow out of the melt takes continuously and almost instantaneously place, the proposed approximation is not only justified by the phenomenological view of the cutting process but quite obvious. Furthermore, the proposed approach even allows for a consideration of the most sophisticated dependence of the bottom kerf width on several factors and factor–factor interactions. Thus, it is assumed that the simulation provides reliable insights into the gas flow characteristics of the real cutting experiments. Calculated velocity distributions, as shown in Figure 13 for the three parameter constellations which correspond to the different cut edge categories according to Figure 4, might be regarded as a confirmation of this assumption. It can be clearly seen that in case of the parameter constellation Run 01 (Category II), the gas flow is separated from the cutting front, and this phenomenon is commonly considered as the principal reason for the occurrence of category II cuts, whereas in case of constellations Run 07 (Category I) and Run 12 (Category III), such a gas flow separation is not observed. By means of the shear stress analysis, it will be shown that even the peculiarities of Category III cuts will be explainable by the gas flow characteristics. Corresponding shear stress distributions at the kerf walls (including the front region) are shown in Figure 14. 

Derived minimum and maximum values of the magnitudes of the shear stress τ_Front,Max_ at the front area, the shear stress τ_Kerf,Max_ at the cut edge and the backward directed component τ_Kerf,X,Max_ as well as the shear stress ratio at the kerf, and also mean values of all quantities are listed in Table 4. Hereby, the values τ_Kerf,Max_ and τ_Kerf,X,Max_ were both determined along the border line at the interface between cut front and cut edge. As it can be derived from Figure 14, the evaluated values of τ_Kerf,Max_ and τ_Kerf,X,Max_ are typically located in the upper region of the kerf.

**Figure 15:**
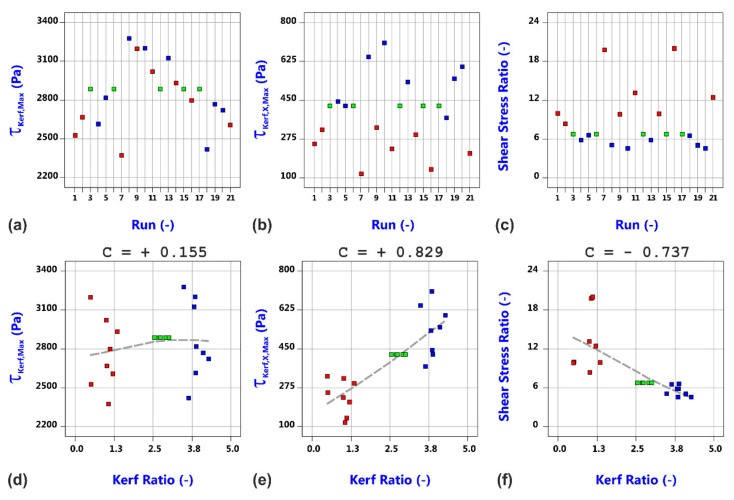
Shear stress data distribution and correlation analysis with respect to the experimentally determined kerf ratio. Data points are colored by the value of the focal plane position (blue: 2.5 mm, green: 5.0 mm, red: 7.5 mm). (**a**) Shear stress maximum vs. run; (**b**) Backward directed shear stress component vs. run; (**c**) Shear stress ratio vs. run; (**d**) Shear stress maximum vs. kerf ratio; (**e**) Backward directed shear stress component vs. kerf ratio; (**f**) Shear stress ratio vs. kerf ratio.

Figure 15 shows the shear stress data distribution and the results of the correlation analysis with respect to the experimentally determined kerf ratio. It is important to note that the calculated τ_kerf,Max_ values (a) are about one order higher than the τ_Kerf,X,Max_ values (b). This conforms to the experimental experience that most of the molten material is blown out downwards. Resultant shear stress ratios lie in the range between 4.5 and 20 (c). 

**Figure 16:**
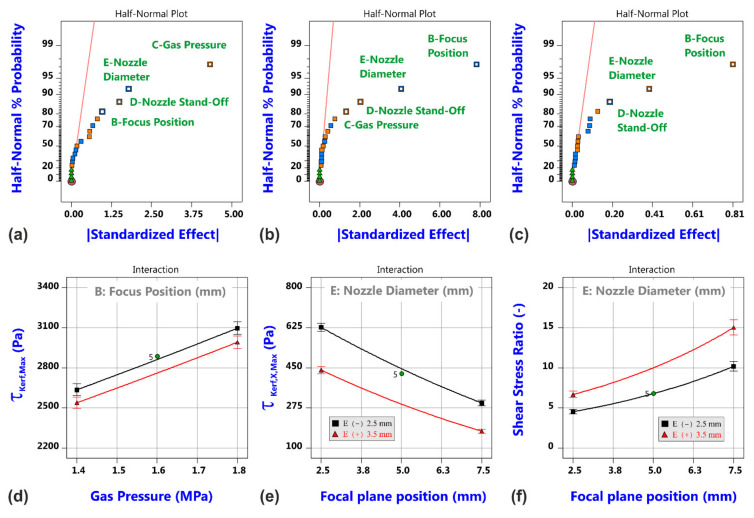
Results of shear stress analysis for the simulation of the cutting gas flow. Half-normal probability plots of shear stress τ_Kerf,Max_ (**a**), shear stress τ_Kerf,X,Max_ (**b**) and shear stress ratio (**c**), and corresponding model graphs (**d**–**f**).

What is particularly interesting to note is the finding that the maximum shear stress τ_Kerf,Max_ does not show any correlation to the kerf ratio whereas the backward directed component of the shear stress as well as the shear stress ratio do (d–f). Hereby, the backward directed component τ_Kerf,X,Max_ strongly increases, and the shear stress ratio accordingly decreases with the kerf ratio. This gives a first decisive link between gas flow characteristics and experimental results, in which all of the Category III cuts with large amounts of lateral melt accumulations were produced in case of high kerf ratios, i.e., for conditions with a strong backward directed shear stress component. Corresponding probability plots and model graphs in Figure 16 clearly show that the varied factors influence the maximum shear stress τ_Max,Kerf_ as well as the backward directed component τ_Max,X,Kerf_ and the stress ratio in a very different way. Therefore, the strength of the magnitude of the shear stress can be easily controlled by addressable gas flow parameters such as the gas pressure, nozzle diameter and nozzle stand-off (a). In contrast, the most vital factor for the backward directed component τ_Kerf,X,Max_ (b) and the shear stress ratio (c) is the focal plane position, i.e., a beam parameter. This means that both quantities are much more influenced by the cut kerf geometry than being controllable by gas parameters. As a result, the generation of Category I cuts within the investigated parameter space at the high focal plane position can be theoretically reasoned by high values of the shear stress magnitude (d), low values of the backward directed shear stress magnitude (e) as well as resultant high shear stress ratios (f). 

**Figure 17:**
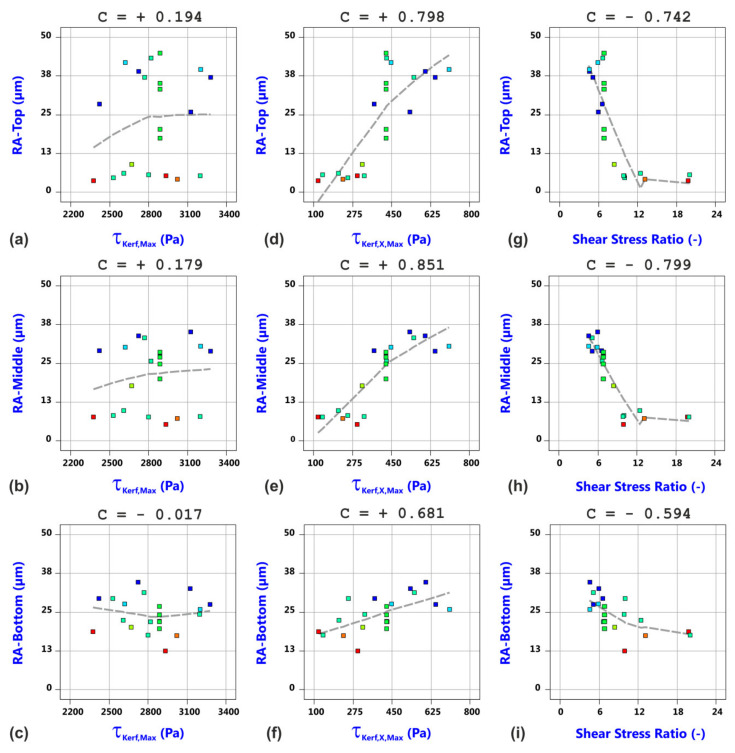
Correlation analysis between experimentally determined cut edge roughness values and computed shear stress values from the gas flow simulation: (**a**) surface roughness top vs. shear stress maximum, (**b**) surface roughness middle vs. shear stress maximum, (**c**) surface roughness bottom vs. shear stress maximum; (**d**) surface roughness top vs. backward directed shear stress component; (**e**) surface roughness middle vs. backward directed shear stress component; (**f**) surface roughness bottom vs. backward directed shear stress component; (**g**) surface roughness top vs. shear stress ratio; (**h**) surface roughness middle vs. shear stress ratio; (**i**) surface roughness bottom vs. shear stress ratio.

In addition, remarkable relationships between the measured cut edge roughness and the calculated shear stress values are found, as shown in Figure 17. It is obvious that the edge roughness does not exhibit any correlation to the magnitude of the shear stress (a–c). This conforms to the process understanding that the principal effect of the shear stress is the downward blow out of the melt and that the melt driven out of the kerf does not contribute to the edge roughness. In contrast, a pronounced correlation is found between the significantly smaller backward component of the shear stress and the edge roughness (d–f). It is consequently concluded that amounts of the melt being forced to flow backward because of these shear stresses contribute to the final kerf edge roughness. Regarding finally the correlation relations between roughness and shear stress ratio, a kind of threshold behavior can be detected (g–i). Roughness values become small if the shear stress ratio is greater than 10.

### 3.3. Factorial Analysis of Beam Oscillation Laser Cutting

Within the purpose of this study, the factorial analysis of beam oscillation laser cutting was focused on a pragmatic evaluation of corresponding effects in comparison to the conventional cutting process. For the sake of simplicity, the omnidirectional circular scan pattern was exemplarily selected to accomplish this task. Despite its two-dimensional shape, this pattern is entirely described in terms of only two parameters, i.e., the oscillation frequency and the oscillation amplitude. To make the results of beam oscillation cutting comparable to the conventional cutting trials, the same levels of both identified vital factors laser power and focal plane position were considered, whereas the secondary gas parameters were kept constant at their center point values, i.e., p_G_ = 1.6 MPa, Δz_N_ = 0.75 mm and d_N_ = 3.0 mm. In combination with the two additional oscillation parameters, a full factorial design could be applied to get again 21 data points including 5 replications at the design center point for the following analysis. Table 5 lists the tested parameter constellations and the corresponding cut edge categories.

**Figure 18:**
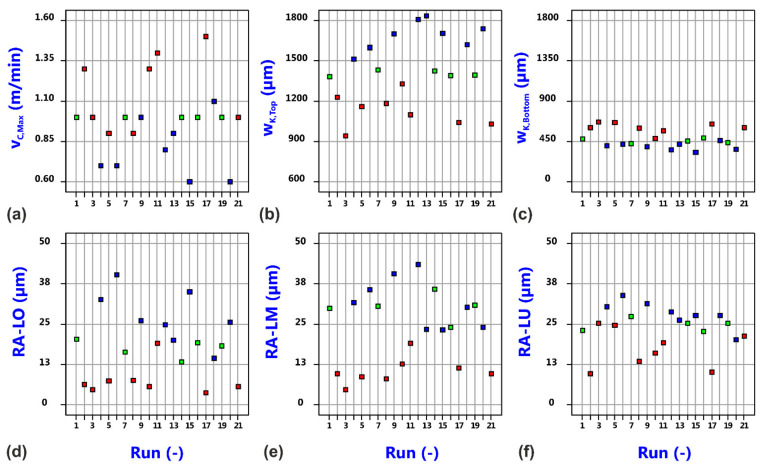
Distribution of raw data of maximum cutting speed (**a**), top kerf width (**b**), bottom kerf with (**c**), top roughness R_a_ (**d**), middle roughness R_a_ (**e**) and bottom roughness (**f**) versus experimental run for laser beam cutting with circular oscillation. The data points are colored by value of focal plane position (Blue: 2.5 mm, Green: 5.0 mm, Red: 7.5 mm).

As before in conventional cutting, all three cut edge categories are present, but the number of quality cuts of Category I is increased from 4 probes in conventional cutting to 7 probes in oscillation cutting. Because of an identical number of Category III cuts for all of the tested constellations at the low and middle focal plane position, this change is related to the accordingly reduced number of Category II cuts. So far, the variation of beam oscillation parameters seems to be more efficient in getting quality cuts than the variation of gas parameters within the investigated parameter ranges. Table 6 lists the minimum, maximum and mean values of the evaluated responses, and Figure 18 shows the raw data distribution versus the run number. It can be stated that the induced changes are again technically significant, whereby—in comparison to the conventional cutting data and with regard to the mean values—(i) the maximum cutting speed is slightly decreased, (ii) the cut kerf width at the top surface as well as the aspect ratio of the kerf are slightly increased, (iii) the kerf width at the bottom surface is dramatically decreased, and (iv) the roughness values do not show a clear tendency and seem to be of similar magnitude in average.

**Figure 19:**
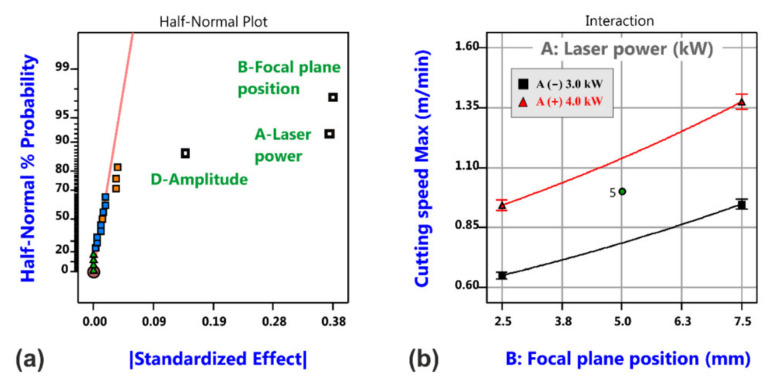
Analysis results of maximum cutting speed for laser cutting with circular beam oscillation. Half-normal probability plot of cutting speed limit (**a**) and corresponding model graph (**b**).

The decrease in maximum cutting speed can be reasoned by the increased interaction area of the oscillating beam in comparison to the static beam. The oscillation amplitude has a negative effect on the maximum cutting speed, i.e., the higher the oscillation amplitude the smaller the cutting speed limit, but the most vital factors remain the focal plane position and the laser power, see Figure 19.

**Figure 20:**
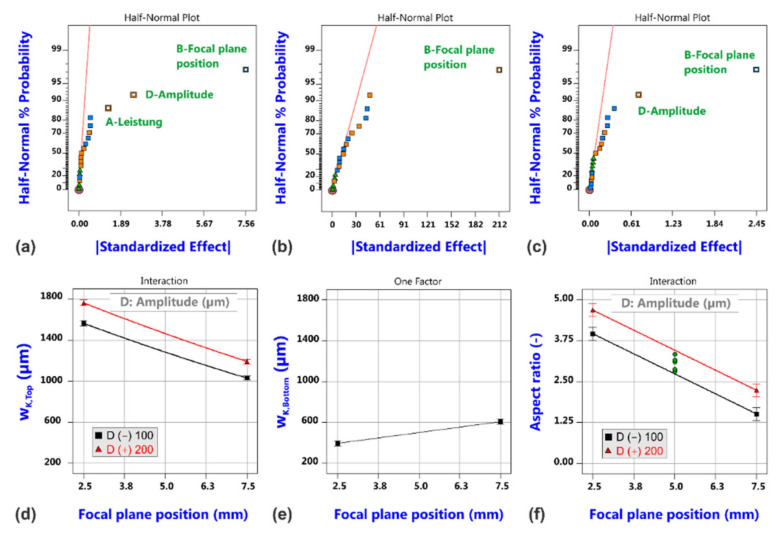
Results of cut kerf geometry analysis for laser cutting with circular beam oscillation. Half-normal probability plots of kerf width w_K,Top_ (**a**), kerf width w_K,Bottom_ (**b**) and aspect ratio (**c**), and corresponding model graphs (**d**–**f**).

Corresponding probability plots and model graphs in Figure 20 demonstrate that the oscillation amplitude also affects the kerf size at the top surface and the kerf ratio (a,c). As might be expectable, increased amplitudes give rise to increased kerf widths (d) and higher aspect ratios (f), and with regard to the kerf width, the effect of the oscillation amplitude is even more pronounced than the effect of laser power. However, the most distinguished change in geometrical kerf characteristics in comparison to the conventional cutting trials is found with respect to the bottom kerf width (b,e). The probability plots only reveal the focal plane position as statistically significant factor, but the model graph shows that the absolute change between both position limits is only small, i.e., the kerf width at the bottom surface remains almost constant within the investigated design space. This surprising phenomenon must be either caused by the lack of gas-related effects on melt flow behavior and kerf formation (gas parameters showed an effect on bottom kerf width in case of the conventional cutting process with variable gas parameters but were kept constant for beam oscillation cutting), or in contrast by a supportive redistribution of energy deposition as a result of the beam oscillation. The last hypothesis is supported by the observation that the large effect of focal plane position on the bottom kerf width in conventional laser cutting is dramatically damped in case of beam oscillation cutting.

**Figure 21:**
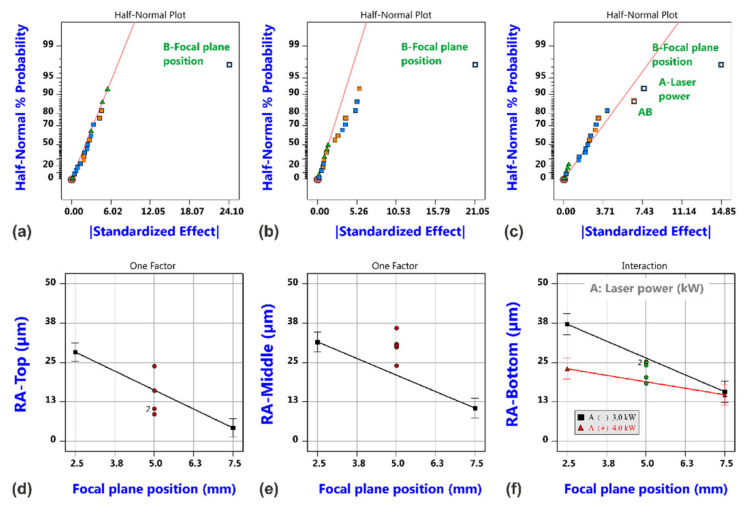
Results of roughness analysis for laser cutting with circular beam oscillation. Half-normal probability plots of surface roughness RA-Top (**a**), RA-Middle (**b**) and RA-Bottom (**c**), and corresponding model graphs (**d**–**f**).

With regard to the edge roughness, the focal plane position is again the most vital factor; see Figure 21. Only the roughness values at the bottom edge region are additionally influenced by the laser power, whereby an interaction indicates that the effect of laser power is only present for the low focal plane position. It is a surprising result that the oscillation parameters are not indicated as statistically significant factors for the cut edge roughness, but this might be a consequence of the fact that the variation in focal plane position caused different cut edge categories and that the corresponding edge appearance including the roughness is dominated by this effect. Nevertheless, the potential of the beam oscillation as promising tool for quality improvements becomes evident if the best probes of both experimental series without and with beam oscillation are compared.

**Figure 22:**
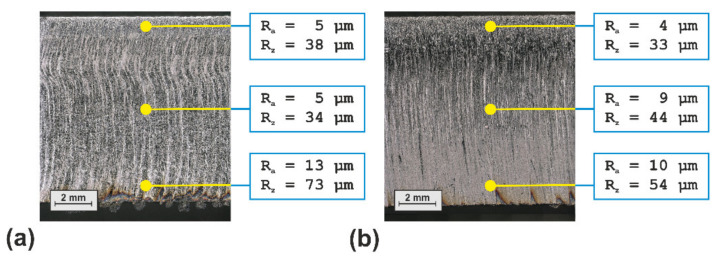
Comparison of the best probes of the performed experimental cutting trials with a static beam ((**a**) Run 14 (Table 2)) and with circular beam oscillation ((**b**) Run 02 (Table 5)).

As can be seen in Figure 22, the cut edge of the best beam oscillation cutting probe shows a more homogeneous striation structure than the best probe of the conventional laser cutting series. The measured roughness values indicate that the roughness range along the kerf edge is reduced from 5–13 µm to 4–10 µm in terms of R_a_ values and from 34–73 µm to 33–54 µm in terms of R_z_ values. Furthermore, the formation of dross at the underside edge of the sheet is almost completely suppressed in case of the beam oscillation cutting probe.

## 4. Summary

Conventional and beam oscillation laser cutting were investigated by factorial designs to reveal the most vital factors on cut performance, cut kerf geometry and cut edge roughness. In addition to the conducted experimental trails, also gas flow simulations were performed to support the interpretation of the experiments.

Under the particular conditions of the performed study, the focal plane position is the most decisive factor with respect to the results of the conventional cutting process, whereas the addressable parameters of the gas flow only show secondary effects on the bottom kerf width and the roughness in the bottom edge region. What is particularly interesting in the results is the finding of noticeable correlations between the measured cut edge roughness and the aspect ratio of the cut kerf geometry.

By combining the experimental design with an adequately designed plan for corresponding gas flow simulations, it could be demonstrated that also essential characteristics of the cut kerf gas flow (in particular the backward directed component of the shear stress as well as the shear stress ratio) are strongly affected by the cut kerf shape. As a result, the occurrence of different cut edge categories can be consistently explained by peculiarities of the gas flow. Furthermore, a detected high correlation between the measured roughness values and the magnitudes of the backward directed shear stress component indicates an inherent dependency of cut edge features on gas flow characteristics. To our best knowledge, such a relationship between shear stress and cut edge roughness was never revealed before.

Most important for the understanding is the finding that the magnitude of the backward directed shear stress component is much more influenced by the kerf geometry than by addressable gas parameters, such as gas pressure, nozzle stand-off distance and nozzle diameter. It means that a careful control of the cut kerf geometry by appropriate laser beam parameters might be regarded as the key for an optimized gas flow to ensure a high quality of the cut edge.

**Figure 23:**
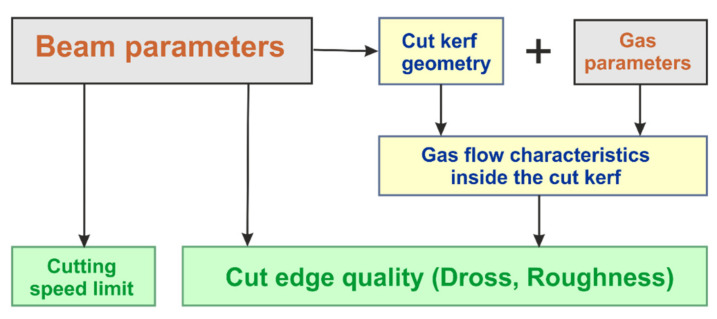
Cause–effect relationships of fiber laser fusion cutting under conditions of the performed study.

Detected relationships between controllable parameters and cutting performance criteria, i.e., cutting speed limit and cut edge quality, are schematically depicted in Figure 23. Within the investigated parameter space of this study, the laser parameters are vital for the achievable cutting speed limit. They also determine the cut kerf geometry. Cut kerf geometry and controllable gas parameters are crucial for the gas flow characteristics inside the cut kerf. Whereas the controllable parameters gas pressure, nozzle diameter and nozzle stand-off distance determine the magnitude of the shear stress acting in axial direction for blowing out the molten material, the most decisive effect for the magnitude of the backward directed shear stress component results from the cut kerf geometry. This backward directed component seems to influence the cut edge roughness directly. New research indicates that the cut edge structure itself might be particularly affected by the hidden structure of the gas flow boundary layer [51,52].

Additional control variables to affect the kerf geometry are addressable for the variant of beam oscillation cutting. In case of the exemplarily investigated circular beam oscillation process, the oscillation amplitude has a direct effect on the top kerf width and the kerf aspect ratio as well. However, more important is the finding that the dependency between bottom kerf width and focal plane position is dramatically changed in comparison to the conventional cutting trials. As a result, the bottom kerf width remains almost constant within the investigated parameter space. This result can be regarded as an implication that some hidden physical mechanisms are acting in beam oscillation cutting which are able to change the characteristics of the cutting process. Those mechanisms being able to influence the direction and strength of the melt film flow in consequence of a modified energy deposition and a changed thermal state of the melt film might involve effects of (i) a changed gas-melt film interaction in dependence on the temperature distribution of the processing zone or (ii) changed fluid dynamical properties (surface tension, viscosity).

## Figures and Tables

**Table 1:** Chemical composition of the AISI 304 stainless steel sheets.

Element	M%
Carbon (C)	0.019
Silicon (Si)	0.556
Manganese (Mn)	1.042
Phosphorous (P)	0.0296
Sulphur (S)	0.0255
Chromium (Cr)	18.206
Nickel (Ni)	8.103
Molybdenum (Mo)	0.398
Cobalt (Co)	0.106
Nitrogen (N)	0.081
Copper (Cu)	0.432
Iron (Fe)	Balance

**Table 2:** Experimental design of the factorial study of conventional laser cutting with a static beam.

Run	Factor A	Factor B	Factor C	Factor D	Factor E	Category
	Laser Power	Focal Plane Position	Cutting Gas Pressure	Nozzle Stand-Off	Nozzle Diameter	
-	(kW)	(mm)	(MPa)	(mm)	(mm)	-
1	3.0	7.5	1.4	0.50	2.5	2
2	4.0	7.5	1.4	1.00	2.5	2
3	3.5	5.0	1.6	0.75	3.0	3
4	4.0	2.5	1.4	1.00	3.5	3
5	4.0	2.5	1.8	0.50	3.5	3
6	3.5	5.0	1.6	0.75	3.0	3
7	4.0	7.5	1.4	0.50	3.5	1
8	3.0	2.5	1.8	0.50	2.5	3
9	3.0	7.5	1.8	1.00	2.5	2
10	4.0	2.5	1.8	1.00	2.5	3
11	4.0	7.5	1.8	1.00	3.5	2
12	3.5	5.0	1.6	0.75	3.0	3
13	3.0	2.5	1.8	1.00	3.5	3
14	4.0	7.5	1.8	0.50	2.5	1
15	3.5	5.0	1.6	0.75	3.0	3
16	3.0	7.5	1.8	0.50	3.5	1
17	3.5	5.0	1.6	0.75	3.0	3
18	3.0	2.5	1.4	0.50	3.5	3
19	4.0	2.5	1.4	0.50	2.5	3
20	3.0	2.5	1.4	1.00	2.5	3
21	3.0	7.5	1.4	1.00	3.5	1

**Table 3:** Raw data of the cutting trials with a static laser beam.

Response	Unit	Minimum	Maximum	Ratio	Mean
Cutting Speed Max	m/min	0.70	1.60	2.28	1.05
Kerf width Top	µm	800	1650	2.06	1218
Kerf width Bottom	µm	816	1685	2.06	1240
Aspect ratio	-	0.48	4.28	8.92	2.5
RA-LO (R_a_ (left side, top))	µm	4	45	11.2	23
RA-LM (R_a_ (left side, middle))	µm	5	35	7.00	21
RA-LU (R_a_ (left side, bottom))	µm	13	35	2.69	24
RA-RO (R_a_ (right side, top))	µm	4	52	13.0	22
RA-RM (R_a_ (right side, middle))	µm	5	35	7.00	17
RA-RU (R_a_ (right side, bottom))	µm	11	37	3.36	24
RZ-LO (R_z_ (left side, top))	µm	23	218	9.48	117
RZ-LM (R_z_ (left side, middle))	µm	34	168	4.94	107
RZ-LU (R_z_ (left side, bottom))	µm	73	176	2.41	122
RZ-RO (R_z_ (right side, top))	µm	25	216	8.64	101
RZ-RM (R_z_ (right side, middle))	µm	31	163	5.26	92
RZ-RU (R_z_ (right side, bottom))	µm	59	181	3.07	118

**Table 4:** Raw data of the simulation of the cutting gas flow.

Response	Unit	Minimum	Maximum	Ratio	Mean
Shear stress τ_Front,Max_ (Front Max)	Pa	2194	3402	1.55	2757
Shear stress τ_Kerf,Max_ (Kerf Max)	Pa	2375	3277	1.38	2834
X Shear stress τ_Kerf,X,Max_ (Kerf Max)	Pa	120	708	5.91	395
Shear stress ratio τ_Kerf,Max_/τ_Kerf,X,Max_	-	4.52	20.0	4.42	9
Shear stress τ_Front,Mean_ (Front Mean)	Pa	1722	2578	1.50	2135
Shear stress τ_Kerf,Mean_ (Kerf Mean)	Pa	1840	2750	1.49	2272
X Shear stress τ_Kerf,X,Mean_ (Kerf Mean)	Pa	51	449	8.84	242

**Table 5:** Experimental design of the factorial study of laser cutting with circular beam oscillation.

Run	Factor A	Factor B	Factor C	Factor D	Cut Edge Category
	Laser Power	Focal Plane Position	Oscillation Frequency	Oscillation Amplitude	
-	(kW)	(mm)	(Hz)	(µm)	-
1	3.5	5.0	2000	150	3
2	4.0	7.5	2800	200	1
3	3.0	7.5	2800	100	2
4	3.0	2.5	2800	100	3
5	3.0	7.5	1200	200	1
6	3.0	2.5	1200	100	3
7	3.5	5.0	2000	150	3
8	3.0	7.5	2800	200	1
9	4.0	2.5	2800	100	3
10	4.0	7.5	1200	200	1
11	4.0	7.5	1200	100	1
12	4.0	2.5	2800	200	3
13	4.0	2.5	1200	200	3
14	3.5	5.0	2000	150	3
15	3.0	2.5	2800	200	3
16	3.5	5.0	2000	150	3
17	4.0	7.5	2800	100	1
18	4.0	2.5	1200	100	3
19	3.5	5.0	2000	150	3
20	3.0	2.5	1200	200	3
21	3.0	7.5	1200	100	1

**Table 6:** Raw data of the cutting trials with circular beam oscillation.

Response	Unit	Minimum	Maximum	Ratio	Mean
Cutting Speed Max	m/min	0.60	1.50	2.50	0.99
Kerf width Top	µm	944	1836	1.94	1408
Kerf width Bottom	µm	331	671	2.03	491
Aspect ratio	-	1.41	5.15	3.65	3.10
RA-LO (R_a_ (left side, top))	µm	4	40	10.0	17
RA-LM (R_a_ (left side, middle))	µm	5	43	8.60	23
RA-LU (R_a_ (left side, bottom))	µm	10	34	3.40	23
RA-RO (R_a_ (right side, top))	µm	3	38	12.7	16
RA-RM (R_a_ (right side, middle))	µm	5	38	7.60	20
RA-RU (R_a_ (right side, bottom))	µm	10	41	4.10	23
RZ-LO (R_z_ (left side, top))	µm	26	170	6.54	89
RZ-LM (R_z_ (left side, middle))	µm	31	217	7.00	117
RZ-LU (R_z_ (left side, bottom))	µm	56	160	2.86	121
RZ-RO (R_z_ (right side, top))	µm	21	187	8.90	78
RZ-RM (R_z_ (right side, middle))	µm	30	206	6.87	107
RZ-RU (R_z_ (right side, bottom))	µm	54	193	3.57	117

## Data Availability

Data sharing is not applicable to this article.
